# Plant-based dietary patterns are associated with slower epigenetic aging

**DOI:** 10.18632/aging.206362

**Published:** 2026-03-19

**Authors:** Hyunju Kim, Christina A. Castellani, Jiantao Ma, Alexis C. Wood, Audrey Ting, Morgan E. Grams, Bing Yu, Kelly Ruggles, James S. Floyd, Dan E. Arking, Casey M. Rebholz

**Affiliations:** 1 Department of Epidemiology, University of Washington, Seattle, WA 98105, USA; 2 Cardiovascular Health Research Unit, Department of Medicine, University of Washington, Seattle, WA 98105, USA; 3 Department of Pathology and Laboratory Medicine, Schulich Medicine & Dentistry, Western University, Ontario N6A5C1, Canada; 4 Department of Epidemiology and Biostatistics, Schulich Medicine & Dentistry, Western University, Ontario NA5C1, Canada; 5 Division of Nutritional Epidemiology and Data Science, Friedman School of Nutrition Science and Policy, Tufts University, Boston, MA 02111, USA; 6 USDA/ARS Children’s Nutrition Research Center, Baylor College of Medicine, Houston, TX 77030, USA; 7 Department of Epidemiology, Johns Hopkins Bloomberg School of Public Health, Baltimore, MD 21205, USA; 8 Division of Precision Medicine, New York University Grossman School of Medicine, New York, NY 10016, USA; 9 Department of Epidemiology, University of Texas Health Sciences Center at Houston School of Public Health, Houston, TX 77030, USA; 10 Department of Medicine, New York University Grossman School of Medicine, New York, NY 10016, USA; 11 Department of Medicine, University of Washington, Seattle, WA 98105, USA; 12 Department of Genetic Medicine, Johns Hopkins University School of Medicine, Baltimore, MD 21205, USA; 13 Welch Center for Prevention, Epidemiology, and Clinical Research, Johns Hopkins University, Baltimore, MD 21205, USA; 14 Department of Medicine, Johns Hopkins School of Medicine, Baltimore, MD 21205, USA

**Keywords:** plant-based diets, DNA methylation, epigenetic aging, all-cause mortality, middle-aged adults

## Abstract

Greater adherence to plant-based diets is associated with health benefits. Dietary intake can modify DNA methylation patterns, but it is unknown whether plant-based diets in a largely non-vegetarian population are associated with DNA methylation-based epigenetic aging measures. We examined the associations between 4 different types of plant-based diets indices (PDI) [overall PDI, provegetarian diet, healthy PDI, and unhealthy PDI] and epigenetic aging. We used data from the Atherosclerosis Risk in Communities (ARIC) Study (N=2,810) and National Health and Nutrition Examination Survey (NHANES, N=2,056). PDIs negatively scored higher intake of animal products and positively scored higher intake of all or selected plant foods (overall PDI and provegetarian diet), healthy plant foods (healthy PDI), and unhealthy plant foods (unhealthy PDI). Associations were examined with GrimAge version2, HannumAge, and PhenoAge in each study. Estimates were meta-analyzed using fixed effects model. Each standard deviation (SD) higher in the overall PDI, provegetarian diet, and healthy PDI was associated with decelerated GrimAge2 (range of *β* = -0.28 to -0.16, P for all tests <0.05). Higher overall PDI and provegetarian diet was associated with decelerated PhenoAge and HannumAge (overall PDI only). No significant association was observed for unhealthy PDI. Following diets rich in plant foods and low in animal products may slow biological aging.

## INTRODUCTION

Plant-based diets are rich in plant foods and low in animal products. Epidemiological studies have found that the quality of plant-based diets may be associated with a lower risk of chronic disease [1–4]. In the Atherosclerosis Risk in Communities (ARIC) Study, we found that greater alignment with overall plant-based diets and provegetarian diets (diets relatively higher in plant foods and lower in animal products) was associated with a lower risk of all-cause mortality and incident cardiovascular disease (CVD) [5]. Healthy plant-based diets (diets relatively higher in healthy plant foods, but lower in unhealthy plant foods and animal products) were associated with a lower risk of cardiovascular risk factors, and deaths due to CVD [3, 6, 7]. In contrast, unhealthy plant-based diets (diets relatively higher in unhealthy plant foods, and lower in healthy plant foods and animal products) were associated with higher risk of these conditions [3, 7].

Epigenetic aging refers to the biological age estimated from DNA methylation markers [8]. Acceleration in epigenetic aging at certain biomarkers, including 1) GrimAge (version1 and version2), 2) Hannum, and 3) PhenoAge [9–12] can represent future risk of morbidity and mortality, independent of chronological age [13, 14]. Studies suggested that certain foods and nutrients (tea, vegetables (e.g., red beetroot), olive oil, and nutrients such as catechin, betalains, lycopene, quercetin, resveratrol, sulforaphane, curcumin, omega-3 fatty acid, and hydroxytyrosol oleic acid) could contribute to healthy aging [15–19]. These foods and nutrients are generally higher in plant-based diets. Thus, improvements in health and longevity associated with plant-based diets could be reflected in DNA methylation-derived aging measures.

Limited data exist on overall dietary patterns on epigenetic aging. Prior studies focused on diet quality, such as the Dietary Approaches to Stop Hypertension (DASH) diet [20], Healthy Eating Index (HEI)-2015, Alternative HEI-2010, DASH diet, and alternate Mediterranean diet (aMED) [21], epigenetic nutrient index (ENI) [22], or vegan diets (exclusion of animal products) [23, 24] and found that individuals with greater adherence to these diets had decelerated epigenetic aging. However, it is unknown if diets relatively higher in plant foods and relatively lower in animal products in a non-vegetarian population are associated with epigenetic aging, or whether the association differs by healthy or unhealthy versions of plant-based diets. This is relevant given that reduction, rather than exclusion of animal products, may be easier to implement in the general population, and opposite associations between healthy and unhealthy versions of plant-based diets and health outcomes have been observed in prior studies [1, 3, 7, 25–28].

We aimed to evaluate the associations between 4 different types of plant-based diet indices (PDI) and 3 measures of epigenetic aging [GrimAge version 2 (GrimAge2), PhenoAge, and HannumAge], leveraging data from the ARIC Study and the National Health and Nutrition Examination Survey (NHANES).

## RESULTS

The mean age in the ARIC Study and NHANES was 57 years and 63 years, respectively. The mean DNA methylation-based aging measures ranged from 52 to 69 years across the two studies. Chronological age was adjusted by calculating the residuals of DNA methylation-based aging measures after regressing each aging metric on chronological age. More than half of the participants in each study were women (Table 1). Two-thirds of the ARIC participants were Black and NHANES participants were non-white. The proportion of current drinkers was higher in NHANES than in the ARIC Study (62% vs. 39%).

**Table 1:** Characteristics of participants in the Atherosclerosis Risk in Communities (ARIC) Study and National Health and Examination Survey (NHANES).^1^

	**ARIC study**	**NHANES**
Sample size (Unweighted), N	2810	2056
Age, years^2^	57.3 (0.1)	62.7 (0.3)
Sex, %		
Men	36.7	47.0
Women	63.3	53.0
Race/ethnicity,^3^ %		
Non-Hispanic Black	66.6	20.6
Non-Hispanic White	33.4	40.4
Mexican American and Other Hispanic	0	35.6
Other race	0	3.4
Education, %		
< High school	27.8	24.6
High school graduate	33.7	27.0
At least some college	38.5	48.3
Smoking status, %		
Never smoker	45.0	43.4
Former smoker	37.3	40.2
Current smoker	17.7	16.5
Physical activity index^4^	2.4 (0.01)	6.3 (0.5)
Total energy intake, kcal/d	1579 (11.0)	1949 (24.5)
Alcohol use, %		
Never drinker	34.9	14.9
Former drinker	25.3	23.2
Current drinker	39.4	62.0
Body mass index, kg/m^2^	28.9 (0.1)	28.6 (0.2)
Overall PDI	49.9 (0.2)	48.1 (0.1)
Provegetarian diet	32.5 (0.1)	32.9 (0.1)
Healthy PDI	50.7 (0.1)	48.6 (0.2)
Unhealthy PDI	49.5 (0.1)	47.6 (0.2)
GrimAge2, years	68.4 (0.1)	69.1 (0.3)
HannumAge, years	67.0 (0.2)	64.1 (0.3)
PhenoAge, years	53.1 (0.2)	52.4 (0.4)

^1^Values are mean (standard error) for continuous variables and proportions for categorical variables. All estimates from NHANES took into account its complex survey design.

^2^Chronological age at visit 2 was reported for participants with DNA methylation data at visit 2 and chronological age at visit 3 was reported for participants with DNA methylation data at visit 3.

^3^In the ARIC Study, ethnicity was not reported, thus we cannot be certain that participants were Non-Hispanic.

^4^Physical activity in the ARIC Study represents activity (range 1-5) after incorporating information on intensity, frequency, and duration of sports-related activities. Physical activity in NHANES represents metabolic equivalents of tasks per minute.

PDI, plant-based diet index.

**Figure 1:**
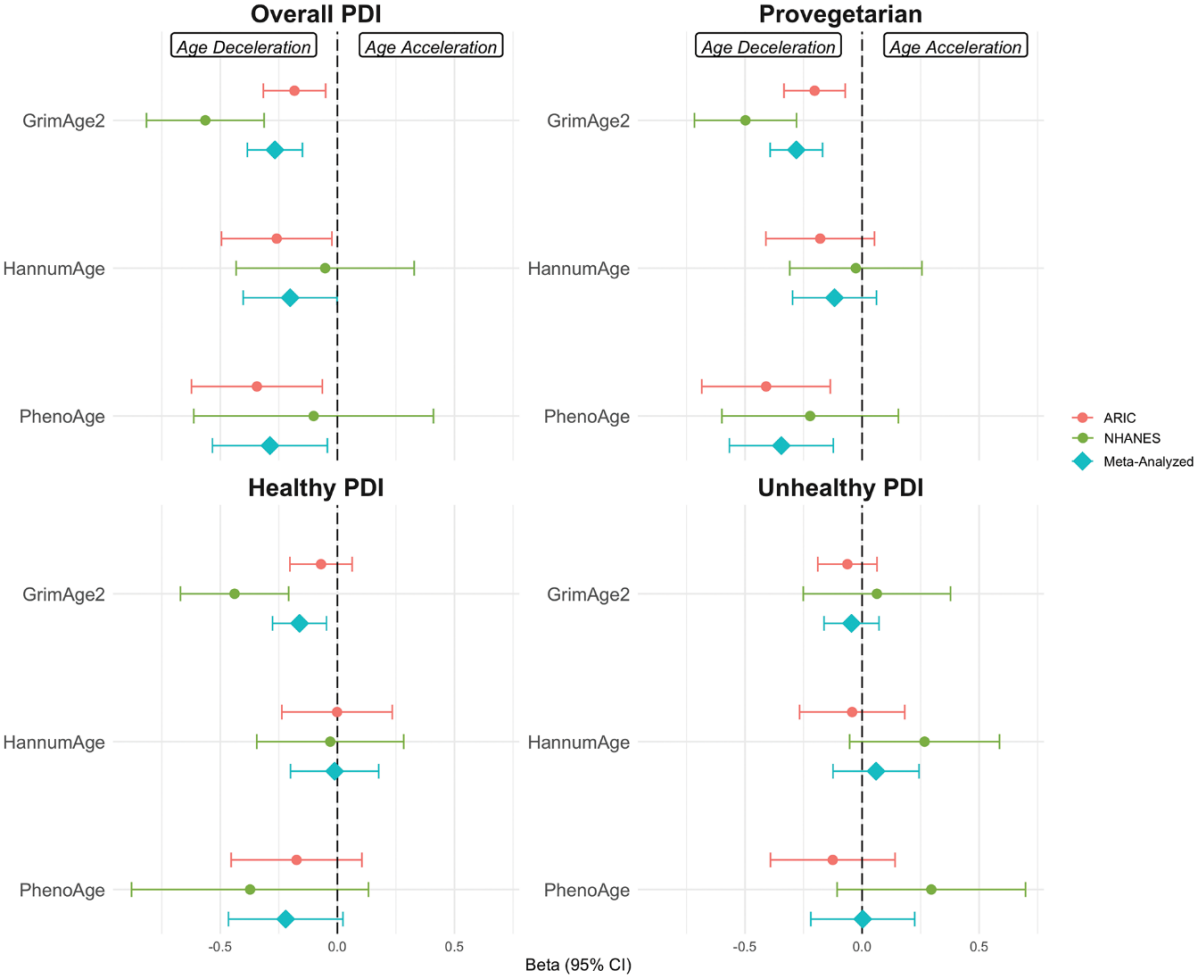
**Associations between different types of plant-based diet indices (PDI) and epigenetic aging in the Atherosclerosis Risk in Communities Study (ARIC) and National Health and Nutrition Examination Survey (NHANES).** Multivariable linear regression model was adjusted for age, sex, race (in ARIC, we used race-center), total energy intake, education, income (NHANES only), smoking status, physical activity, alcohol intake, and margarine intake (ARIC only). Estimates were meta-analyzed using fixed-effects model. In NHANES, survey-weighted linear regression was used (weighted N= 64,294,854). PDI, plant-based diet index, CI, confidence interval.

After adjusting for sociodemographic characteristics, dietary factors, and health behaviors, greater adherence to the overall PDI and provegetarian diet was associated with GrimAge2 and PhenoAge deceleration (Figure 1 and Supplementary Table 1). Each standard deviation (SD) higher in the overall PDI, provegetarian diet, and healthy PDI was associated with 0.16 to 0.28 slower GrimAge2 (meta-analyzed *β*_overall PDI_,-0.27, 95% CI: -0.38, -0.15; *β*_provegetarian diet_,-0.28, 95% CI: -0.39, -0.17; *β*_healthy PDI_,-0.16, 95% CI: -0.28, -0.05, P for all tests<0.05). Each SD higher in the overall PDI was associated with 0.20 slower HannumAge (meta-analyzed *β*, -0.20, 95% CI: -0.40, 0.001). Each SD higher in the overall PDI and provegetarian diet was associated with 0.28 to 0.34 slower PhenoAge (meta-analyzed *β*_overall PDI_,-0.28, 95% CI: -0.39, -0.17; *β*_provegetarian diet_,-0.34, 95% CI: -0.56, -0.12, P for all tests<0.05).

Associations between healthy PDI and GrimAge2 were statistically significant in NHANES (*β*,-0.44, 95% CI: -0.67, 0.21, P<0.001) but not in the ARIC Study (*β*,-0.07, 95% CI: -0.20, 0.06, P=0.30). No significant association was observed for unhealthy PDI and any of the DNA methylation-based aging. Results were similar when BMI was additionally adjusted as a covariate in the model which controlled for sociodemographic characteristics, dietary factors, and health behaviors (Supplementary Table 2).

When food components were simultaneously adjusted, each SD higher intake of healthy plant food (representing 3.3 servings) was associated with reduced GrimAge2 and PhenoAge by -0.095 to -0.128 in meta-analysis (Table 2, Supplementary Table 3). Each SD higher intake of animal products (representing 2.9 servings) was associated with accelerated PhenoAge (*β*, 0.48; 95% CI: 0.19, 0.78, P=0.001) in the ARIC Study, but decelerated PhenoAge in NHANES (*β*, -0.14; 95% CI: -0.22, -0.05, P=0.004).

**Table 2:** Associations between per standard deviation higher intake of food components within plant-based diets and epigenetic aging in the Atherosclerosis Risk in Communities (ARIC) Study and National Health and Examination Survey (NHANES).^*^

	***β* (95% Confidence intervals)**		
	**GrimAge2**	**HannumAge**	**PhenoAge**
ARIC (N=2,810)			
Healthy plant food	-0.109 (-0.158, 0.060)	-0.039 (-0.110, 0.032)	-0.080 (-0.153, -0.008)
Unhealthy plant food	-0.003 (-0.009, 0.003)	-0.006 (-0.014, 0.001)	-0.004 (-0.013, 0.006)
Animal product	-0.028 (-0.105, 0.050)	-0.046 (-0.125, 0.033)	0.483 (0.191, 0.776)
NHANES (N=2,056)			
Healthy plant food	-0.429 (-0.622, -0.236)	-0.155 (-0.434, 0.125)	-0.317 (-0.604, -0.030)
Unhealthy plant food	-0.143 (-0.429, 0.143)	-0.298 (-0.669, 0.072)	-0.182 (-0.636, 0.272)
Animal product	-0.068 (-0.257, 0.121)	-0.113 (-0.306, 0.080)	-0.139 (-0.224, -0.047)
Meta-Analyzed			
Healthy plant food	-0.128 (-0.174, -0.083)	-0.046 (-0.112, 0.020)	-0.095 (-0.162, -0.027)
Unhealthy plant food	-0.003 (-0.009, 0.003)	-0.006 (-0.014, 0.001)	-0.004 (-0.013, 0.005)
Animal product	-0.033 (-0.102, 0.035)	-0.056 (-0.126, 0.014)	-0.088 (-0.169, -0.007)

^*^Multivariable linear regression model was adjusted for age, sex, race (in ARIC, we used race-center), total energy intake, education, income (NHANES only), smoking status, physical activity, alcohol intake, and margarine intake (ARIC only). Estimates were meta-analyzed using fixed-effects model. In NHANES, survey-weighted linear regression was used (weighted N= 64,294,854). All food components within plant-based diets were adjusted simultaneously.

In mediation analyses, GrimAge2 mediated 33% to 42% of the associations between the overall PDI, provegetarian diet, and healthy PDI, and all-cause mortality (P for all tests <0.001) (Table 3). PhenoAge was not a significant mediator (P for all tests >0.05).

**Table 3:** Mediation of epigenetic aging for the association between plant-based diet indices and all-cause mortality in the Atherosclerosis Risk in Communities (ARIC) Study and National Health and Examination Survey (NHANES).^*^

	**Proportion mediated (95% confidence interval) by GrimAge2**	**P-value**	**Proportion mediated (95% confidence interval) by PhenoAge**	**P-value**
**NHANES (N=2,056)**				
Overall PDI	36.7 (28.1, 43.2)	<0.001	2.4 (-67.7, 33.8)	0.92
Provegetarian	32.7 (24.5, 39.4)	<0.001	6.5 (-17.3, 22.3)	0.51
Healthy PDI	41.9 (31.9, 49.9)	<0.001	-	-
Unhealthy PDI	-	-	-	-

^*^Model was adjusted for age, sex, race-center, total energy intake, education, income, smoking status, physical activity, alcohol intake. Analyses were not conducted in the ARIC Study because there was no significant association between plant-based diets and all-cause mortality in this subsample.

- indicates that no significant association was observed for the specific dietary pattern and all-cause mortality or epigenetic aging measures. Therefore, the proportion mediated was not valid (greater than 100%).

PDI, plant-based diet index.

In sensitivity analyses, the results did not change meaningfully when we additionally adjusted for cell composition (Supplementary Table 4). However, the association between healthy PDI and GrimAge2 was no longer statistically significant (meta-analyzed *β*, -0.09, 95% CI: -0.22, 0.03). When all food groups were adjusted together, higher intake of whole grain was consistently associated with decelerated GrimAge2 in both studies (range of *β*=-0.14 to -0.41), and similar associations were observed for intake of fruits and vegetables (range of *β*=-0.21 to -0.41) in NHANES (Supplementary Table 5). Higher intake of animal fat was associated with accelerated GrimAge2 (*β*, 0.17; 95% CI: 0.04, 0.30) in the ARIC Study. These results were largely consistent when each of the 17 food groups were included in the model individually, except that greater intake of sugar-sweetened beverages were additionally associated with accelerated GrimAge2 in NHANES only (*β*, 0.34; 95% CI: 0.15, 0.53). When ARIC participants were restricted to those in visit 3, all estimates had the same direction of association as the main results, but the results were attenuated except for the association between unhealthy PDI and HannumAge (Supplementary Table 6). Specifically, a 1-SD higher in unhealthy PDI was associated with HannumAge acceleration of 0.701 (95% CI: 0.149, 1.254).

In post-hoc analysis, we conducted a stratified analysis by physical activity (<median, ≥median). We found that the associations between plant-based diets and epigenetic aging were more pronounced among those with higher physical activity levels (i.e., greater adherence to the overall PDI, provegetarian diet, and healthy PDI was associated with decelerated GrimAge2 in NHANES; Supplementary Table 7). Interestingly, a 1-SD higher in unhealthy PDI was associated with HannumAge and PhenoAge acceleration of 0.095 to 0.133 among those with higher physical activity levels in NHANES.

## DISCUSSION

The present study found that dietary patterns higher in plant foods and lower in animal products were consistently associated with decelerated DNA methylation-derived aging biomarkers, specifically GrimAge2 and PhenoAge. Greater adherence to healthy plant-based diets was associated with decelerated GrimAge2, but results attenuated in sensitivity analyses. Healthy plant food intake was associated with decelerated GrimAge2 and PhenoAge. In our mediation analysis, GrimAge2 mediated the association between plant-based diets, and all-cause mortality. These results suggest that consuming diets higher in plant foods, especially healthy plant foods, may be a strategy to slow aging.

The findings of our study are broadly consistent with prior studies on diet quality and epigenetic aging. In the Framingham Heart Study (N=1,995), per 1-SD higher in the DASH diet score was associated with -0.07 to -0.09 lower levels of GrimAge version 1 (GrimAge1), PhenoAge and Dunedin Pace of Aging Methylation (DunedinPoAm) [20]. In the Sisters Study of 2,694 women, higher levels of four different dietary indices [HEI-2015, Alternative HEI-2010, DASH diet, and aMED] were associated with reduced PhenoAge and GrimAge1 [21]. Recently, a cross-sectional study of 342 Black and White women of the National Heart, Lung, and Blood Institute Growth and Health Study found similar results as the two prior studies, and reported that higher intake of indices comprised of nutrients (vitamins A, C, E, folate, B12, zinc, selenium, magnesium, the ratio of monounsaturated fat to saturated fat, isoflavones, lower amount of total sugar) was associated with reduced GrimAge2 whereas higher added sugar intake was associated with increased GrimAge2 [22]. We extend these studies by identifying that plant-based diets are associated with decelerated GrimAge2.

The mechanisms through which plant-based diets are associated with slower epigenetic age likely involve reduction of oxidative stress and inflammation. Healthy plant foods are high in dietary fiber, and antioxidants (vitamins C and E, carotenoids, polyphenols), and are low in pro-inflammatory compounds such as saturated fat [7]. Reduced inflammation can prevent changes in DNA methylation patterns that are induced by reactive oxygen species (ROS) [29]. In support of this hypothesis, plant-based diets were inversely associated with inflammatory biomarkers (serum C-reactive protein, fibrinogen, and total leukocyte count) [30, 31]. Additionally, individuals following plant-based diets had lower overall adiposity, better lipid profile (such as lower low-density lipoprotein (LDL)-cholesterol (C)), and lower incidence of incident hypertension [7, 32]. All of these conditions can accelerate biological aging by contributing to excessive production of ROS and oxidative damage, which can lead to cellular dysfunction and cell death [33, 34]. Furthermore, recent studies using Mendelian randomization analysis found evidence that blood lipid profile causally impacted DNA methylation [35–37]. Given this directionality, favorable changes in blood lipids might mediate the association between plant-based diets and epigenetic aging. Nevertheless, it is worth noting that healthy plant-based diet, which emphasizes high quality plant foods, was not significantly associated with GrimAge2 in the ARIC Study but was associated with decelerated GrimAge2 in NHANES, and associations attenuated after adjustment of blood cell composition. In our analyses of food groups, several components of healthy plant foods (whole grains, fruits, vegetables) were associated with reduced GrimAge2 in NHANES, but not in ARIC, which might explain these associations. Therefore, whether a healthy plant-based diet is associated with GrimAge2 requires confirmation in an independent population.

The present study found that GrimAge2 and PhenoAge were associated with more dietary exposures than HannumAge. Whereas overall plant-based diets and provegetarian diets were associated with both decelerated GrimAge2 and PhenoAge, and healthy plant-based diets were additionally associated with decelerated GrimAge2, only overall plant-based diets were inversely associated with HannumAge. Our findings that dietary exposures might be more related to GrimAge2 are in line with prior studies of dietary patterns [HEI-2015, Alternative HEI-2010, DASH diet, and aMED] that reported associations with only GrimAge1 or GrimAge2 [20–22]. PhenoAge was developed to predict aging-related mortality outcomes [10], but HannumAge was developed to predict chronological age [9]. GrimAge1 was developed as a weighted linear combination of smoking, age, sex, and 7 plasma proteins (inferred from cytosine methylation levels) [12]. GrimAge version 2 was updated from GrimAge1 by adding two new proteins (high sensitive C-reactive protein and hemoglobin A1c) estimated from DNA methylation [11]. Healthful dietary patterns can lower systematic inflammation and play a central role in preventing and managing hyperglycemia, which might explain the consistent associations we observed with GrimAge2 [38–41]. Our findings highlight the importance of considering differences in each of these metrics when assessing the association between dietary exposures and DNA methylation-based aging measures. Further, the present study raises a possibility that cytosine-phosphate-guanine (CpG) sites selected for GrimAge2 may be more relevant for reflecting mechanisms underlying plant-based diets and healthy aging. GrimAge2 might be considered for evaluating potential anti-aging effects of dietary exposures.

We did not find evidence that an unhealthy plant-based diet was associated with age acceleration. In our prior study, ARIC participants with greater adherence to unhealthy plant-based diets had higher added sugar intake [7]. Given the prior results which found that per gram higher intake of added sugar was associated with 0.02 accelerated GrimAge2 [22], we expected to find a positive association between unhealthy plant-based diets and GrimAge2. However, in this prior study, the association between added sugar intake and GrimAge2 represented a small difference (0.02 higher GrimAge2 per 1 gram of added sugar) [22]. The present study was larger (N>2000 each for the ARIC Study and NHANES), which included men, and had more ethnically and geographically diverse participants. In the future, it would be informative to investigate whether the effect of unhealthy plant foods on epigenetic clock is less pronounced than that of healthy plant foods, and assess whether added sugars modify epigenetic aging.

Strengths of the present study include the use of two large population-based studies which included diverse groups of middle-aged adults, and use of established plant-based diet indices which will allow comparisons with different populations in the future.

Several limitations are worth noting. The timing of dietary assessment and DNA methylation profiling was not concordant for some participants in the ARIC Study. However, in the ARIC Study, differences in dietary intake over a 6-year period (visit 1 and visit 3) were small [42] and average of visit 1 and visit 3 intake data improves the precision of usual dietary intake estimation. Further, the direction of association was consistent in a sensitivity analysis when the analytic sample was restricted to those from ARIC visit 3. Additionally, the associations were stronger for some diet-epigenetic age associations (healthy plant-based diets-GrimAge2) in NHANES where timing of dietary assessment and DNA methylation profiling was concordant. Second, self-reported dietary intake is subject to measurement and systematic biases. However, in the ARIC Study, trained interviewers administered FFQ with estimates for portion sizes, which reduces measurement error. Third, although our analyses adjusted for important confounders (physical activity, alcohol consumption, smoking), we cannot rule out the possibility of residual confounding. Fourth, the ARIC Study used Illumina 450K array while NHANES used EPIC array. There may be coverage differences across CpG sites for the calculation of epigenetic aging measures. It may be informative to compare CpG sites common between the two arrays, and re-estimate epigenetic aging measures. However, EPIC array data are not available in the ARIC Study, and NHANES does not provide individual CpG site level data. In the future, it may be worth directly comparing the associations between plant-based diets and common CpG sites between the two arrays. Lastly, diet was assessed at one time point (average of two time points for some participants in the ARIC Study). Thus, we were not able to establish causality on dietary habits and epigenetic age deceleration.

We found that overall plant-based diets, provegetarian diets, and healthy plant-based diets were associated with slower GrimAge2, possibly reflecting the role of these dietary patterns on healthy aging. Additionally, GrimAge2 mediated the association between plant-based diets and mortality, which highlights that DNA methylation may be an important mediator for diet-lifespan associations.

## MATERIALS AND METHODS

### Study design and study population

The ARIC Study is an ongoing prospective cohort study which enrolled 15,792 middle-aged predominantly black and white men and women (45-64 years of age) in 1987-1989 (visit 1) from four U.S communities (Washington County, Maryland; Forsyth County, North Carolina; Minneapolis, Minnesota; Jackson, Mississippi) [43]. Participants attended in-person visits [visit 2 (1990-1992) and visit 3 (1993-1995)]. DNA methylation data were available at visit 2 or visit 3 in a subset of predominantly African American participants from Jackson, Mississippi, and Forsyth County, North Carolina. Each study center reviewed the protocol and approved the study.

The National Health and Nutrition Examination Survey (NHANES) is a biennial survey which uses a multi-stage, stratified, clustered sampling design to examine the health and nutritional status of non-institutionalized US population [44]. We used the NHANES 1999-2000 and 2001-2002 cycles where DNA methylation data were available among those aged ≥50 years [45]. Participants completed one 24-hour dietary recall, provided biospecimens, and underwent physical examinations at the mobile examination center. The National Center for Health Statistics’ Research Ethics Review Board approved the study. All participants in the ARIC Study and NHANES provided informed consent.

In ARIC, participants were selected for DNA methylation if they agreed to use of their DNA, available DNA was at least >1 μg, and if there were genome-wide genotyping data available. We excluded participants without DNA methylation data, those with implausible dietary intake, missing covariates, missing plant-based diets. The analytic sample was 2,810 participants for the ARIC Study (Supplementary Figure 1).

In NHANES, participants aged ≥50 years with available biospecimens were eligible for DNA methylation assessment [45]. Then, a random sample of non-Hispanic white (approximately 50%) and a random sample of the remaining racial groups (Mexican American, Other Hispanic, Non-Hispanic White, Non-Hispanic Black, and other race) were selected for DNA methylation assessment. We applied similar exclusion criteria as the ARIC Study, and additionally excluded participants aged ≥85 years and older, because their chronological age was provided as 85 years old to protect the privacy of the participants. The unweighted sample size was 2,056 participants and weighted sample size was 64,294,854 for the NHANES (Supplementary Figure 2).

### Plant-based diet indices

At visit 1 and visit 3 of the ARIC Study, trained interviewers administered a modified 66-item Willett food frequency questionnaire (FFQ) where participants reported the frequency with which they consumed foods and beverages of a defined serving size in the past year. Reproducibility of this FFQ was quantified in the ARIC study, and the Willett FFQ was validated [46–48]. Dietary intake at visit 1 and visit 3 was averaged. Despite the fact that dietary intake and DNA methylation was not measured contemporaneously, a previous study in the ARIC Study found that differences in diet between visit 1 and visit 3 was minimal [42].

All indices were analyzed as continuous variables (per standard deviation [SD] higher). Calculation of plant-based diet indices in the ARIC Study has been previously published [3]. Briefly, all foods and beverages on the FFQ were classified into one of 17 food groups for the overall PDI, healthy PDI, and unhealthy PDI, and 11 food groups for the provegetarian diet index [25, 49]. For the overall PDI, higher intake of all plant foods received higher scores (positive scoring). For the healthy PDI, higher intake of only plant foods considered healthy received higher scores. Healthy plant foods included whole grains, fruits, vegetables, nuts and seeds, legumes, tea and coffee. For the unhealthy PDI, higher intake of unhealthy plant foods received higher scores. Unhealthy plant foods included refined grains, potatoes, sugar-sweetened beverages, fruit juices, and sweets and desserts. We followed the original PDI to classify plant foods healthy vs. unhealthy. In the development of PDI, healthy and unhealthy plant foods were determined based on prior studies of food group and disease associations [4, 25]. Plant foods that had mixed associations with disease outcomes in prior studies (e.g., alcohol) were not included in the indices and adjusted as a covariate. The provegetarian diet index had a similar scoring scheme as the overall PDI in that all plant foods received positive scoring but did not include several food groups (fruit juices, sweets and desserts). In all of these scores, higher intake of animal products (animal fat, dairy, eggs, fish or seafood, meat, and miscellaneous animal products) received lower scores (reverse scoring). For each food group, the possible range was from 1 to 5.

The same scoring scheme was used for 24-hour recall data in NHANES. In NHANES, miscellaneous animal products were excluded from animal products, because foods in the miscellaneous animal products (e.g., lasagna) have already been accounted for in other animal product categories (e.g., meat, eggs, dairy). All individual foods reported in the 24-hour dietary recall were converted to MyPyramid Equivalents Database (MPED) [50]. For food groups that were not available in MPED (e.g., coffee and tea, fruit juices, sweets and desserts, animal fats), we manually identified individual food items by reading the list of food descriptions in the 24-hour dietary recalls after.

### Covariates

In the ARIC Study, participants reported demographic characteristics (age, sex, race, education), smoking, and physical activity. Participants could report race from four categories (White, Black, American Indian or Alaskan Indian, or Asian or Pacific Islander). Physical activity was assessed using the Baecke questionnaire and was represented with a score of 1 to 5 after incorporating information on intensity, frequency, and duration of sports-related activities [51]. Other dietary factors (total energy intake, alcohol intake, margarine intake) were calculated from the FFQ. Margarine intake was adjusted as a covariate only in the ARIC study, because it was not clear if it should be a healthy plant food or unhealthy plant food [4] and to be consistent with prior studies of plant-based diets in ARIC participants [3, 5, 7]. Trained staff measured height and weight, which was used to calculate body mass index (BMI). In the ARIC study, we used covariates measured at the time of blood draw (visit 2 or visit 3).

In NHANES, participants reported similar demographic characteristics. Participants self-reported race and ethnicity as Mexican American, other Hispanic, Non-Hispanic White, Non-Hispanic Black, and other race. Physical activity was calculated as metabolic equivalent of tasks (MET) after considering daily activities, leisure-time activities, and sedentary activities [52]. Similar to the ARIC Study, trained staff measured height and weight in participants of NHANES which was used to derive BMI. Income was considered a covariate in NHANES as a ratio of family income to poverty threshold. Education was used as a proxy for socioeconomic status in the ARIC Study, given that a large proportion of participants had missing data on income.

### Epigenetic aging

In the ARIC Study, DNA methylation measurement was conducted using whole blood leukocyte specimens using the Gentra Puregene Blood Kit (Qiagen, Valencia, CA, USA). Genomic DNA was bisulfite converted using the EZ-96 DNA Methylation Kit (Zymo Research, Irvine, CA, USA). Then, the Illumina Infinium HumanMethylation450 Beadchip array and Illumina GenomeStudio were used to quantify DNA methylation (Illumina, San Diego, CA, USA) [53, 54]. The 450K array can identify more than 450,000 CpG sites. Beta values (ratio of methylated to overall signal) were used to represent methylation score for each CpG site. NOOB was used for background correction and the beta mixture quantile (BMIQ) method was used to normalize DNA methylation data.

In NHANES, DNA was extracted from whole blood, bisulfite converted, and DNA methylation was measured using the Illumina Infinium MethylationEPIC Beadchip v1.0 (Illumina, San Diego, CA, USA). The EPIC array can identify more than 850,000 CpG sites. Details on quality control, preprocessing steps, and outlier removal have been reported previously [45]. Briefly, color correction was conducted using a set of control probes which allowed for determination of system background intensity levels. Specimens were removed if the median intensity value of both the methylated and unmethylated values were <10.5. Then, probes that had more than 5% of missing data were imputed according to the method recommended by the creators of epigenetic clocks [9–11]. DNA methylation data were normalized using the beta mixture quantile (BMIQ) method and corrected for probe type bias [55]. In both studies, we calculated residuals of DNA methylation-based aging metrics after adjusting for chronological age in linear regression models. Blood cell type composition was estimated using the Houseman equation in both studies using R packages (the minfi in the ARIC Study [56] and FlowSorted.Blood.EPIC in NHANES [57]).

### Statistical analysis

Characteristics of the study participants were summarized using means and standard errors for continuous variables and proportions for categorical variables. All analyses in NHANES used survey weights to consider its complex survey design.

To examine the association between plant-based diets and epigenetic aging, we conducted multivariable linear regression models using each PDI as the predictor and each of the residuals of epigenetic aging metrics as the response variable, adjusting for age, sex, race, education, income (in NHANES only), smoking, physical activity, and dietary factors (total energy intake, alcohol intake, margarine intake). In the ARIC Study, we combined race and study center variables due to non-uniform distribution of racial groups across the study centers. Models were applied in each study and estimates were meta-analyzed using fixed-effects model. Fixed-effects model was used given that studies report that estimates from random-effects models are imprecise for a small number of studies [58, 59]. Additionally, we repeated the main analyses further adjusting for BMI.

As secondary analyses, we examined if specific components of plant-based diets (healthy plant foods, unhealthy plant foods, and animal products) were important for the association between plant-based diets and GrimAge2 by adjusting for all of these components simultaneously.

Then, we conducted a mediation analysis to estimate the degree to which epigenetic aging mediates the association between plant-based diets and all-cause mortality [60]. The first model estimated the association between plant-based diets and epigenetic aging, adjusting for covariates described above. Then, Cox proportional hazard model was conducted, additionally adjusting for epigenetic aging as a potential mediator. We reported proportion mediated and associated P-values for GrimAge2 and PhenoAge, two epigenetic clocks that were consistently associated with plant-based diets. This analysis was conducted only in NHANES, because there was no significant association between plant-based diets and all-cause mortality in this analytic sample of the ARIC Study.

As sensitivity analyses, we 1) additionally adjusted for blood cell composition (CD4+ T cells, B cells, natural killer cells, and neutrophils estimated from DNA methylation data), 2) simultaneously and individually adjusted for 17 food groups within the overall PDI, healthy PDI, and unhealthy PDI using GrimAge2 as the response variable, and 3) examined the association between plant-based diets and epigenetic aging among only ARIC participants (n=506) who had concordant timing of dietary assessment and DNA methylation. For the analyses on 17 food groups within the overall PDI, healthy PDI, and unhealthy PDI, we focused on GrimAge2 because GrimAge2 was the only metric that was inversely associated with the overall PDI, provegetarian diet, and healthy PDI. All analyses were conducted in R version 4.5.0 (Vienna, Austria).

Lastly, as a post-hoc analysis, we reported our primary analysis stratified by physical activity levels (< median and ≥median). Meta-analyses were not conducted because physical activity assessments differ between the ARIC Study [51] and NHANES [52].

## Supplementary Material

Supplementary Figures

Supplementary Tables
