# Supplementary material for: Plant-based dietary patterns are associated with slower epigenetic aging
Source: Aging (Albany NY). 2026 Mar 19;18(1):138–58. doi: 10.18632/aging.206362 (PMC13249531; doi:10.18632/aging.206362)
Supplement: Supplementary Figures [file aging-18-1-206362-s001.pdf]

SUPPLEMENTARY FIGURES

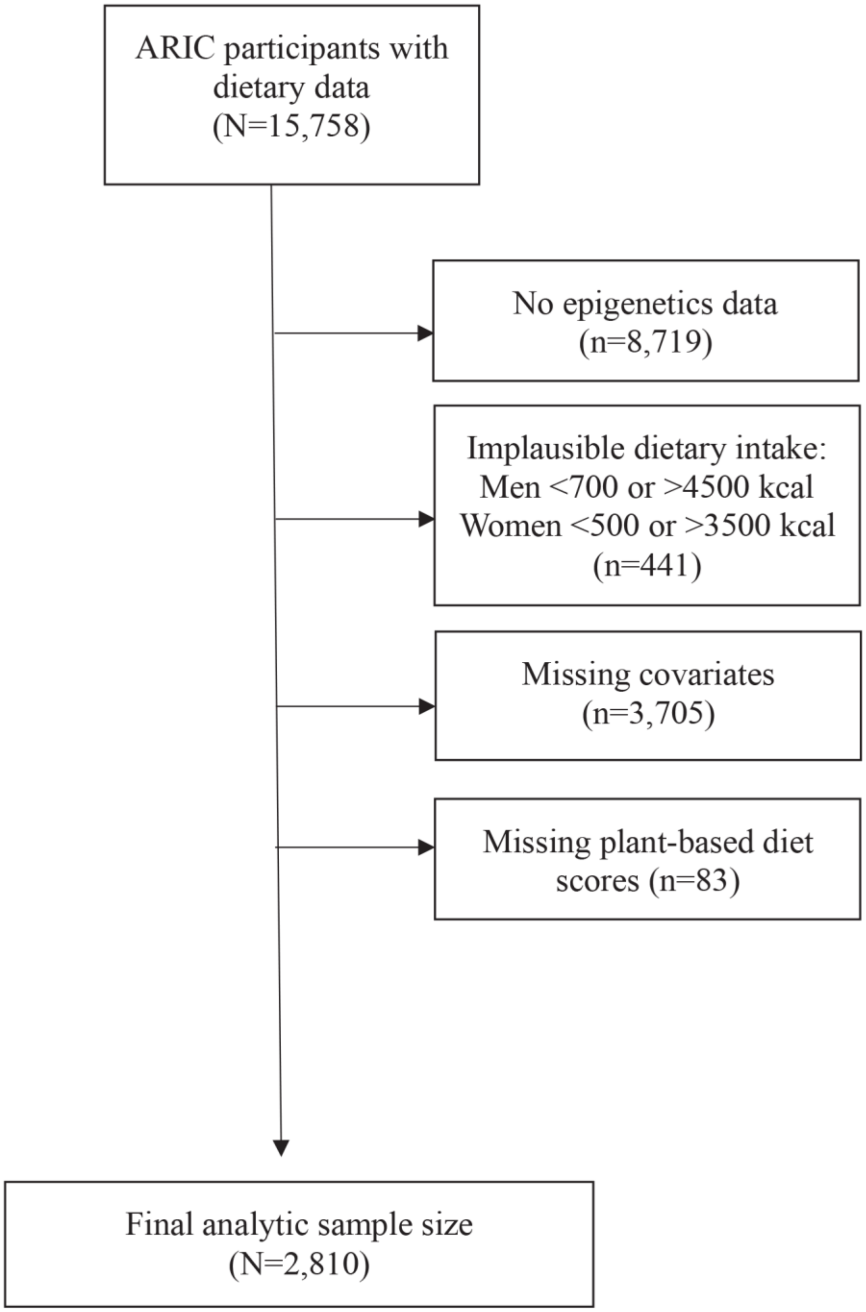

Supplementary Figure 1. Flow diagram of study participants in the Atherosclerosis Risk in Communities (ARIC) study.

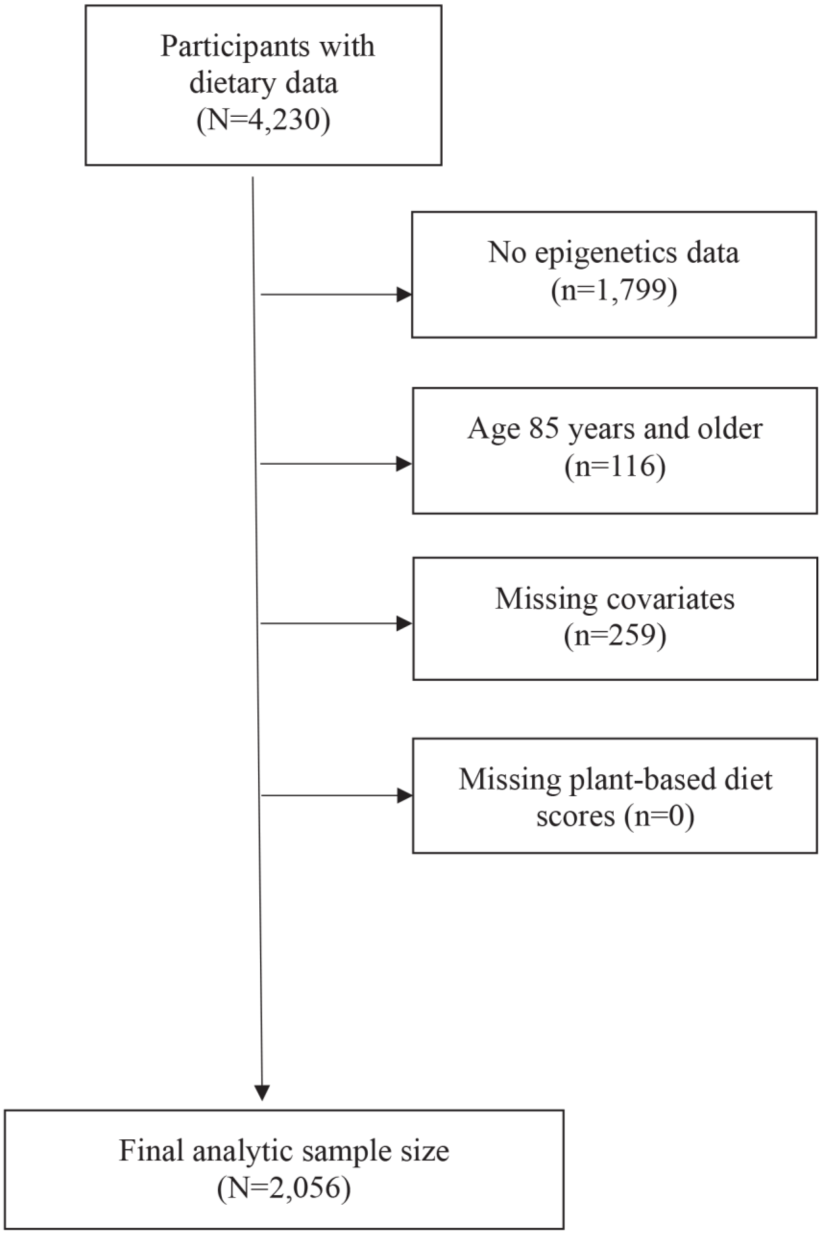

Supplementary Figure 2. Flow diagram of study participants in the National Health and Nutrition Examination Survey (NHANES).
