# Supplementary material for: Plant-based dietary patterns are associated with slower epigenetic aging
Source: Aging (Albany NY). 2026 Mar 19;18(1):138–58. doi: 10.18632/aging.206362 (PMC13249531; doi:10.18632/aging.206362)
Supplement: Supplementary Tables [file aging-18-1-206362-s002.pdf]

SUPPLEMENTARY TABLES

Supplementary Table 1. Associations between plant-based diet indices and epigenetic aging in the Atherosclerosis Risk in Communities (ARIC) study (N=2,810) and the National Health and Nutrition Examination Survey (NHANES, N=2,056).*

	β (95% Confidence intervals)		
	**GrimAge2**	**HannumAge**	**PhenoAge**
**ARIC Study (N=2,810)**			
Overall PDI	-0.183 (-0.316, -0.050)	-0.259 (-0.495, -0.023)	-0.340 (-0.623, -0.064)
Provegetarian	-0.202 (-0.333, -0.072)	-0.179 (-0.410, 0.052)	-0.410 (-0.684, -0.135)
Healthy PDI	-0.070 (-0.202, 0.062)	-0.001 (-0.237, 0.233)	-0.174 (-0.453, 0.104)
Unhealthy PDI	-0.062 (-0.189, 0.063)	-0.042 (-0.266, 0.182)	-0.125 (-0.391, 0.140)
**NHANES (N=2,056)**			
Overall PDI	-0.564 (-0.815, -0.312)	-0.052 (-0.432, 0.327)	-0.101 (-0.613, 0.410)
Provegetarian	-0.498 (-0.716, -0.280)	-0.026 (-0.308, 0.255)	-0.221 (-0.599, 0.155)
Healthy PDI	-0.439 (-0.670, -0.208)	-0.030 (-0.344, 0.283)	-0.373 (-0.879, 0.133)
Unhealthy PDI	0.063 (-0.251, 0.377)	0.266 (-0.052, 0.586)	0.295 (-0.106, 0.698)
**Meta-Analyzed**			
Overall PDI	-0.267 (-0.384, -0.149)	-0.201 (-0.402, -0.001)	-0.288 (-0.533, -0.042)
Provegetarian	-0.280 (-0.392, -0.168)	-0.117 (-0.296, 0.0615)	-0.344 (-0.566, -0.123)
Healthy PDI	-0.161 (-0.277, -0.046)	-0.012 (-0.200, 0.176)	-0.220 (-0.465, 0.023)
Unhealthy PDI	-0.045 (-0.162, 0.072)	0.059 (-0.124, 0.2433)	0.002 (-0.219, 0.224)

*Multivariable linear regression model was adjusted for age, sex, race (in ARIC, we used race-center), total energy intake, education, income (NHANES only), smoking status, physical activity, alcohol intake, and margarine intake (ARIC only). Estimates were meta-analyzed using fixed-effects model. In NHANES, survey-weighted linear regression was used (weighted N= 64,294,854).
PDI, plant-based diet index.

Supplementary Table 2. Associations between plant-based diet indices and epigenetic aging, with body mass index as a covariate in the Atherosclerosis Risk in Communities (ARIC) Study and the National Health and Nutrition Examination Survey (NHANES).*

	β (95% Confidence Intervals)		
	**GrimAge2**	**HannumAge**	**PhenoAge**
**ARIC Study (N=2,810)**			
Overall PDI	-0.189 (-0.322, -0.056)	-0.2488 (-0.484, -0.012)	-0.327 (-0.606, -0.048)
Provegetarian	-0.213 (-0.344, -0.082)	-0.160 (-0.392, 0.072)	-0.380 (-0.654, -0.105)
Healthy PDI	-0.077 (-0.210, 0.055)	0.0139 (-0.221, 0.249)	-0.150 (-0.430, 0.128)
Unhealthy PDI	-0.066 (-0.192, 0.060)	-0.0388 (-0.263, 0.185)	-0.115 (-0.381, 0.150)
**NHANES (N=2,056)**			
Overall PDI	-0.516 (-0.778, -0.255)	-0.032 (-0.404, 0.338)	-0.051 (-0.562, 0.459)
Provegetarian	-0.465 (-0.696, -0.234)	-0.013 (-0.292, 0.265)	-0.188 (-0.569, 0.192)
Healthy PDI	-0.407 (-0.656, -0.158)	-0.017 (-0.330, 0.294)	-0.341 (-0.863, 0.180)
Unhealthy PDI	0.096 (-0.216, 0.409)	0.280 (-0.041, 0.602)	0.329 (-0.077, 0.735)
**Meta-Analyzed**			
Overall PDI	-0.256 (-0.375, -0.137)	-0.186 (-0.385, 0.012)	-0.264 (-0.509, -0.018)
Provegetarian	-0.274 (-0.388, -0.161)	-0.099 (-0.278, 0.078)	-0.314 (-0.537, -0.092)
Healthy PDI	-0.151 (-0.268, -0.033)	0.002 (-0.185, 0.190)	-0.193 (-0.439, 0.052)
Unhealthy PDI	-0.043 (-0.160, 0.074)	0.065 (-0.118, 0.249)	0.017 (-0.204, 0.240)

* Multivariable linear regression model was adjusted for age, sex, race (in ARIC, we used race-center), total energy intake, education, income (NHANES only), smoking status, physical activity, alcohol intake, margarine intake (ARIC only), and body mass index (BMI). Estimates were meta-analyzed using fixed-effects model. In NHANES, survey-weighted linear regression was used (weighted N=64,294,854).
PDI, plant-based diet index.

Supplementary Table 3. Associations between per serving higher in food components within plant-based diets and epigenetic aging in the Atherosclerosis Risk in Communities (ARIC) Study and National Health and Examination Survey (NHANES).*

	β (95% Confidence Intervals)		
	**GrimAge2**	**HannumAge**	**PhenoAge**
**ARIC (N=2,810)**			
Healthy plant food	0.0254 (-0.0184, 0.0692)	-0.0520 (-0.1297, 0.0256)	-0.0470 (-0.1390, 0.0448)
Unhealthy plant food	-0.0086 (-0.0729, 0.0557)	0.0367 (-0.0772, 0.1507)	0.0051 (-0.1298, 0.1400)
Animal product	0.0305 (-0.0267, 0.0877)	0.0793 (0.126, -0.0221)	0.1986 (0.0784, 0.3187)
**NHANES (N=2,056)**			
Healthy plant food	-0.0349 (-0.0505, -0.0192)	-0.0125 (-0.0352, 0.0101)	-0.0258 (-0.0491, -0.0024)
Unhealthy plant food	-0.0014 (-0.0043, 0.0014)	-0.0030 (-0.0067, 0.0007)	-0.0018 (-0.0064, 0.0027)
Animal product	-0.0113 (-0.0432, 0.0204)	-0.0190 (-0.0515, 0.0134)	-0.0560 (-0.0922, -0.0197)
**Meta-Analyzed**			
Healthy plant food	-0.0285 (-0.0428, -0.0143)	-0.0155 (-0.0364, 0.0055)	-0.0270 (-0.0487, -0.0052)
Unhealthy plant food	-0.0015 (-0.0042, 0.0013)	-0.0030 (-0.0066, 0.0006)	-0.0018 (-0.0062, 0.0026)
Animal product	-0.0021 (-0.0290, 0.0248)	-0.0105 (-0.0403, 0.0192)	-0.0363 (-0.0697, -0.0030)

*Multivariable linear regression model was adjusted for age, sex, race (in ARIC, we used race-center), total energy intake, education, income (NHANES only), smoking status, physical activity, alcohol intake, and margarine intake (ARIC only). Estimates were meta-analyzed using fixed-effects model. In NHANES, survey-weighted linear regression was used (weighted N=64,294,854).

Supplementary Table 4. Sensitivity analysis additionally adjusting for blood cell composition the Atherosclerosis Risk in Communities (ARIC) Study and the National Health and Nutrition Examination Survey (NHANES).

	β **(95% Confidence Intervals)**		
	**GrimAge2**	**HannumAge**	**PhenoAge**
**ARIC (N=2,810)**			
Overall PDI	-0.162 (-0.310, -0.014)	-0.182 (-0.419, 0.055)	-0.147 (-0.467, 0.173)
Provegetarian	-0.163 (-0.311, -0.014)	-0.109 (-0.348, 0.128)	-0.292 (-0.612, 0.028)
Healthy PDI	-0.021 (-0.171, 0.129)	0.112 (-0.127, 0.352)	-0.025 (-0.348, 0.298)
Unhealthy PDI	-0.078 (-0.217, 0.062)	-0.240 (-0.463, 0.015)	-0.248 (-0.548, 0.052)
**NHANES (N=2,056)**			
Overall PDI	-0.432 (-0.696, -0.167)	-0.143 (-0.489, 0.205)	-0.012 (-0.482, 0.458)
Provegetarian	-0.392 (-0.620, -0.163)	-0.193 (-0.494, 0.109)	-0.179 (-0.559, 0.200)
Healthy PDI	0.038 (-0.259, 0.336)	0.122 (-0.219, 0.463)	0.193 (-0.212, 0.597)
Unhealthy PDI	0.096 (-0.216, 0.409)	0.280 (-0.041, 0.602)	0.329 (-0.077, 0.735)
**Meta-Analyzed**			
Overall PDI	-0.230 (-0.358, -0.102)	-0.168 (-0.361, 0.024)	-0.101 (-0.362, 0.158)
Provegetarian	-0.234 (-0.357, -0.112)	-0.143 (-0.327, 0.040)	-0.246 (-0.483, -0.003)
Healthy PDI	-0.092 (-0.218, 0.034)	0.011 (-0.163, 0.187)	-0.087 (-0.351, 0.175)
Unhealthy PDI	-0.055 (-0.180, 0.069)	-0.103 (-0.304, 0.097)	-0.083 (-0.319, 0.154)

*Multivariable linear regression model was adjusted for age, sex, race (in ARIC, we used race-center), total energy intake, education, income (NHANES only), smoking status, physical activity, alcohol intake, margarine intake (ARIC only), body mass index (BMI) and blood cell composition. Estimates were meta-analyzed using fixed-effects model. In NHANES, survey-weighted linear regression was used (weighted N=64,294,854).
PDI, plant-based diet index.

Supplementary Table 5. Associations between 17 food groups (per standard deviation higher) within the overall plant-based diet index (PDI), healthy PDI, unhealthy PDI and GrimAge2 in the Atherosclerosis Risk in Communities (ARIC) Study and the National Health and Nutrition Examination Survey (NHANES).*

	β (95% Confidence Intervals)			
	Adjustment of food groups simultaneous		Food groups adjusted separately	
	ARIC (N=2,810)	NHANES (N=2,056)	ARIC (N=2,810)	NHANES (N=2,056)
Whole grains	**-0.140 (-0.272, -0.008)**	**-0.414 (-0.610, -0.218)**	**-0.134 (-0.261, -0.007)**	**-0.414 (-0.603, -0.226)**
Fruits	-0.064 (-0.214, 0.084)	**-0.412 (-0.643, -0.182)**	-0.018 (-0.149, 0.112)	**-0.343 (-0.532, -0.154)**
Vegetables	0.057 (-0.113, 0.229)	**-0.210 (-0.400, -0.020)**	0.084 (-0.044, 0.212)	**-0.215 (-0.402, -0.028)**
Nuts and seeds	-0.047 (-0.181, 0.087)	-0.145 (-0.336, 0.045)	-0.066 (-0.192, 0.059)	-0.139 (-0.325, 0.046)
Legumes	0.062 (-0.095, 0.220)	0.004 (-0.188, 0.197)	0.082 (-0.044, 0.208)	0.030 (-0.157, 0.217)
Tea and coffee	0.012 (-0.085, 0.220)	-0.101 (-0.291, 0.088)	-0.011 (-0.139, 0.100)	-0.113 (-0.302, 0.076)
Refined grains	0.053 (-0.079, 0.186)	-0.230 (-0.431, 0.028)	0.070 (-0.057, 0.198)	-0.155 (-0.341, 0.031)
Potatoes	-0.043 (-0.188, 0.101)	0.070 (-0.118, 0.260)	-0.009 (-0.140, 0.121)	0.119 (-0.065, 0.304)
Sugar-sweetened or artificially sweetened beverages	0.042 (-0.087, 0.171)	0.171 (-0.029, 0.371)	0.034 (-0.093, 0.160)	**0.339 (0.149, 0.529)**
Fruit juices	-0.021 (-0.151, 0.108)	0.138 (-0.084, 0.361)	-0.019 (-0.145, 0.106)	-0.063 (-0.248, 0.122)
Sweets and desserts	-0.093 (-0.230, 0.042)	-0.166 (-0.358, 0.026)	-0.074 (-0.020, 0.052)	-0.082 (-0.269, 0.104)
Animal fat	**0.167 (0.036, 0.298)**	-0.001 (-0.186, 0.184)	**0.019 (0.051, 0.307)**	-0.029 (-0.214, 0.157)
Dairy	0.059 (-0.076, 0.196)	-0.082 (-0.279, 0.114)	0.064 (-0.065, 0.195)	-0.079 (-0.267, 0.108)
Eggs	-0.024 (-0.166.117)	-0.029 (-0.217, 0.158)	0.088 (-0.042, 0.218)	0.062 (-0.123, 0.248)
Fish or seafood	0.070 (-0.063, 0.205)	-0.094 (-0.288, 0.100)	0.045 (-0.081, 0.173)	-0.059 (-0.245, 0.127)
Meat	0.062 (-0.090, 0.214)	-0.065 (-0.276, 0.145)	0.029 (-0.104, 0.162)	0.132 (-0.054, 0.318)
Miscellaneous animal products	-0.070 (-0.215, 0.074)	-	-0.024 (-0.154, 0.104)	-

*Multivariable linear regression model was adjusted for age, sex, race (in ARIC, we used race-center), total energy intake, education, income (NHANES only), smoking status, physical activity, alcohol intake, and margarine intake (ARIC only). Miscellaneous animal products were not included for NHANES, because foods in the miscellaneous animal products (e.g., lasagna) have already been accounted for in other animal product categories (e.g., meat, eggs, dairy). Mean variance inflation factor for models that simultaneously adjusted for food groups in ARIC was 1.97 and 1.41 in NHANES. Bold indicates statistically significant associations.
($P < 0.05$). In NHANES, survey-weighted linear regression was used (weighted N= 64,294,854).

Supplementary Table 6. Sensitivity analysis restricting the participants to Atherosclerosis Risk in Communities (ARIC) Study visit 3 (N=506).

	β (95% Confidence Intervals)		
	GrimAge2	HannumAge	PhenoAge
Overall PDI	-0.167 (-0.484, 0.150)	-0.214 (-0.751, 0.324)	-0.362 (-1.024, 0.300)
Provegetarian	-0.210 (-0.526, 0.106)	-0.277 (-0.812, 0.258)	-0.433 (-1.093, 0.22)
Healthy PDI	0.061 (-0.253, 0.375)	-0.149 (-0.681, 0.382)	-0.275 (-0.931, 0.381)
Unhealthy PDI	-0.015 (-0.344, 0.312)	0.701 (0.149, 1.254)	0.091 (-0.594, 0.776)

*Multivariable linear regression model was adjusted for age, sex, race-center, total energy intake, smoking status, physical activity, alcohol intake, and margarine intake.
PDI, plant-based diet index.

Supplementary Table 7. Associations between plant-based diet indices and epigenetic aging, stratified by physical activity levels (<median, ≥median) in the Atherosclerosis Risk in Communities (ARIC) Study and the National Health and Nutrition Examination Survey (NHANES).*

	β (95% Confidence Intervals)		
	**GrimAge2**	**HannumAge**	**PhenoAge**
**ARIC Study (N=2,810)**			
**Physical activity levels <median**			
Overall PDI	-0.131 (-0.319, 0.056)	-0.336 (-0.658, -0.013)	-0.080 (-0.473, -0.312)
Provegetarian	-0.141 (-0.329, 0.046)	-0.185 (-0.508, 0.137)	-0.217 (-0.609, -0.174)
Healthy PDI	-0.035 (-0.232, 0.153)	0.035 (-0.286, 0.358)	-0.179 (-0.571, 0.212)
Unhealthy PDI	-0.037 (-0.209, 0.135)	-0.075 (-0.371, 0.221)	-0.126 (-0.487, 0.233)
**Physical activity levels ≥median**			
Overall PDI	-0.269 (-0.458, -0.082)	-0.245 (-0.590, 0.101)	-0.718 (-1.117, -0.319)
Provegetarian	-0.290 (-0.472, -0.109)	-0.236 (-0.569, 0.096)	-0.692 (-1.075, -0.308)
Healthy PDI	-0.134 (-0.322, 0.053)	-0.092 (-0.435, 0.251)	-0.250 (-0.648, 0.147)
Unhealthy PDI	-0.072 (-0.262, 0.117)	0.021 (-0.327, 0.368)	-0.084 (-0.487, 0.318)
**NHANES (N=2,056)**			
**Physical activity levels <median**			
Overall PDI	-0.091 (-0.173, -0.008)	-0.064 (-0.185, 0.056)	-0.053 (-0.195, 0.088)
Provegetarian	-0.075 (-0.172, 0.022)	-0.056 (-0.168, 0.054)	-0.059 (-0.235, 0.116)
Healthy PDI	-0.006 (-0.098, 0.086)	-0.044 (-0.155, 0.068)	-0.005 (-0.151, 0.145)
Unhealthy PDI	-0.051 (-0.137, 0.034)	-0.024 (-0.122, 0.073)	-0.063 (-0.168, 0.043)
**Physical activity levels ≥median**			
Overall PDI	-0.107 (-0.159, -0.054)	0.016 (-0.101, 0.133)	0.003 (-0.144, 0.151)
Provegetarian	-0.124 (-0.193, -0.056)	0.018 (-0.061, 0.096)	-0.038 (-0.167, 0.091)
Healthy PDI	-0.103 (-0.162, -0.045)	0.0004 (-0.059, 0.066)	-0.103 (-0.209, 0.003)
Unhealthy PDI	0.049 (-0.022, 0.122)	0.095 (0.024, 0.168)	0.133 (0.034, 0.232)

Multivariable linear regression model was adjusted for age, sex, race (in ARIC, we used race-center), total energy intake, education, income (NHANES only), smoking status, alcohol intake, and margarine intake (ARIC only). Meta-analysis was not conducted because different physical activity assessment methods were used in ARIC and NHANES.
